# Comparative language performance in children and adolescents with 22q11.2ds syndrome and down syndrome

**DOI:** 10.1186/s11689-026-09701-4

**Published:** 2026-05-26

**Authors:** Esther Moraleda-Sepúlveda, Miguel Lázaro-López-Villaseñor, Noelia Pulido-García, Hoda Benomar-Simou

**Affiliations:** Department of Psychology, Faculty of Pychology, University Complutense, Campus de Somosaguas, 28223 Pozuelo de Alarcón, Madrid, Spain

**Keywords:** Down Syndrome, Language Development, Linguistic profile, 22q11.2DS Syndrome

## Abstract

Genetically defined neurodevelopmental syndromes provide a framework for examining constraints on language development. This study compared language performance in children and adolescents with 22q11.2 deletion syndrome (22q11.2DS; *n* = 40) and Down syndrome (DS; *n* = 40), matched for age and nonverbal cognitive ability, aged 6–16 years. Standardized assessments included the Clinical Evaluation of Language Fundamentals (CELF-5) and the BLOC-C to evaluate multiple receptive and expressive language domains. Group differences, effect sizes, and associations with age were analyzed to characterize syndrome-specific profiles. Children with 22q11.2DS demonstrated relatively stronger receptive vocabulary and syntax alongside weaker morphosyntactic and pragmatic skills, with vocabulary showing moderate positive associations with age. In contrast, the DS group exhibited generally lower performance across domains, with pronounced difficulties in morphosyntax and limited age-related gains. These findings highlight differences in overall level of performance and relative strengths within a globally impaired profile across syndromes and emphasize the value of multi-dimensional assessment in capturing both age-related patterns and vulnerabilities.

## Introduction

Language development is a complex process involving the interaction of multiple linguistic subsystems, including lexical, morphosyntactic, semantic, and pragmatic skills, which are organized under neurodevelopmental and environmental constraints. Genetically defined neurodevelopmental disorders, such as 22q11.2 deletion syndrome (22q11.2DS) and Down syndrome (DS), provide a unique opportunity to investigate how different genetic variations affect the organization and trajectory of language across childhood and adolescence. Direct comparisons between these populations can identify relative strengths and vulnerabilities that may reflect syndrome-specific constraints on brain development and linguistic modularity. Both 22q11.2DS and DS are associated with intellectual disability and atypical neurodevelopment, although their genetic etiologies differ. 22q11.2DS is caused by a microdeletion on the long arm of chromosome 22 [27], with an estimated prevalence of 1 in 2,000 to 4,000 live births [21]. The syndrome is characterized by high phenotypic variability, including craniofacial anomalies, congenital malformations such as cleft palate [65], and speech-related difficulties associated with structural and neuroanatomical alterations [4, 58]. Cognitive functioning ranges from typical levels to moderate intellectual disability, with approximately 50% of individuals presenting an IQ below 70 [6]. In contrast, DS, the most common genetic cause of intellectual disability [5, 57], results from trisomy 21 [28] and occurs in approximately 1 in 700 live births [3]. It is associated with a characteristic phenotypic profile [17], cognitive impairments affecting memory, attention, and executive functions [24, 31], as well as delays in communicative and linguistic development [20]. These neurocognitive and biological differences are likely to influence the organization of language across domains in each syndrome.

From a lexical and semantic perspective, both groups show delayed vocabulary development; however, important differences have been reported. In 22q11.2DS, several studies have identified relative strengths in receptive vocabulary compared to other linguistic domains [10, 56], suggesting that lexical learning mechanisms may remain relatively preserved and sensitive to environmental input despite broader neurocognitive limitations [7, 8]. In DS, lexical and semantic development is also delayed [9], although comprehension and lexical-semantic skills are often described as relative strengths within the overall language profile [38, 45, 49]. However, vocabulary development in DS appears less influenced by age, with slower growth trajectories and potential plateauing over time [60], suggesting reduced developmental plasticity compared to 22q11.2DS.

In terms of morphosyntactic abilities, both syndromes show clear impairments, although the severity and pattern differ. Individuals with DS consistently exhibit profound morphosyntactic deficits, with syntactic abilities falling well below expectations based on cognitive level and vocabulary [1, 13, 14, 16, 44, 54, 55]. This domain has been widely described as the most affected in DS, with a relatively homogeneous pattern across individuals [22, 26, 35, 41, 42, 47, 62]. In 22q11.2DS, morphosyntactic difficulties are also well documented [51], including impairments in syntactic comprehension and production. However, these deficits often coexist with relatively better performance in vocabulary, contributing to a more uneven linguistic profile. More broadly, expressive language difficulties in 22q11.2DS affect all linguistic components, including phonology, morphosyntax, lexico-semantics, and pragmatics [18, 51], and are frequently accompanied by speech production difficulties such as articulation deficits and hypernasality [4, 30].

Regarding pragmatic and higher-order language skills, both groups experience difficulties, though their nature may differ. In 22q11.2DS, pragmatic impairments are a prominent feature, affecting discourse-level processing, narrative abilities, and social communication [25, 33, 48]. Individuals with this syndrome often produce inadequate communicative acts, provide insufficient contextual information, and show difficulties in interpreting social cues such as facial expressions and emotional states [11, 32, 48]. These challenges significantly impact social relationships [50]. In DS, although pragmatic skills can reach a functional level, deficits have also been reported, including reduced conversational repair strategies and difficulties in maintaining effective communication [36, 37, 39, 40, 64]. However, these difficulties are often interpreted in the context of broader cognitive and linguistic delays, rather than as a clearly dissociated domain.

Taken together, these findings suggest that while both syndromes are characterized by language impairments, the organization of these impairments differs, with DS reflecting a more globally constrained and relatively homogeneous profile, and 22q11.2DS showing a more uneven and dissociated pattern across linguistic domains.

However, despite evidence for these distinct profiles, few studies have directly compared 22q11.2DS and DS using standardized language assessment batteries. Much of the existing literature has examined each syndrome independently, limiting the ability to determine whether observed differences reflect true syndrome-specific profiles or methodological variability across studies. A direct comparative approach across multiple linguistic domains is therefore necessary to clarify similarities and differences in language organization.

In addition, relatively little is known about age–language associations within each syndrome, particularly in relation to vocabulary development. Examining these associations may provide insight into age-related patterns and the degree of plasticity of different linguistic subsystems.

Therefore, the present study aims to:

- Compare language profiles of children with 22q11.2DS and DS across receptive and expressive domains, morphosyntax, semantics, and pragmatics using widely recognized standardized assessments (CELF-5, Peabody Picture Vocabulary Test, and BLOC-C).
- Investigate age–language associations to assess the relative plasticity of lexical versus structural language development in each syndrome.
- Examine patterns of relative strengths and weaknesses in each group, testing whether the groups differed both in overall language level and in the relative distribution of performance across domains.

We hypothesize that:

- H1: Children with 22q11.2DS will demonstrate relative strengths in vocabulary alongside persistent deficits in morphosyntax and pragmatics, whereas children with DS will show a more uniformly impaired profile in structural language domains.
- H2: Lexical performance in 22q11.2DS will show a positive association with age, reflecting continued plasticity, whereas age–performance associations in DS will be weaker or absent.
- H3: We expected that children with 22q11.2DS and children with Down syndrome would differ in overall level of language performance, and we explored whether their patterns across domains would be parallel or show relative differences in strengths and weaknesses

This approach allows us not only to identify descriptive differences but also to interpret the findings in terms of underlying neurocognitive mechanisms and age-related patterns, providing a theoretical framework for understanding the constraints and plasticity associated with each syndrome.

## Method

### Participants

A total of 80 school-aged children and adolescents, aged between 6 and 16 years (Primary and Secondary Education), participated in this study. Of these, 40 individuals were diagnosed with 22q11.2DS Deletion Syndrome, while the remaining 40 had Down Syndrome. Participants were recruited through patient advocacy organizations, clinical databases, and community networks in Spain. Families provided informed consent in accordance with the Declaration of Helsinki, and the study was approved by the Faculty of Health Sciences at the University of Castilla-La Mancha (Code 15/2023).

Inclusion criteria were: (a) genetically confirmed diagnosis of 22q11.2DS or trisomy 21; (b) age between 6 and 16 years; (c) native Spanish speaker; and (d) ability to complete standardized language assessments. Exclusion criteria included: (a) severe uncorrected hearing or vision impairments; (b) uncontrolled epilepsy; and (c) severe psychiatric or neurological disorder preventing test completion.

All participants in both groups were of White ethnicity, with no other ethnic backgrounds represented in the sample. This uniformity in ethnicity was maintained across the 22q11.2DS deletion syndrome group and the Down syndrome group.

The 22q11.2DS deletion syndrome group included 40 children (20 girls and 20 boys) with a mean age of 10.74 years (Standard Deviation = 3.41). The Down syndrome group included 40 children (18 girls and 22 boys) with a mean age of 10.77 years (Standard Deviation = 2.82). Both groups were matched for chronological age. Prior to data collection, to control for cognitive ability, all participants completed the Wechsler Intelligence Scale for Children–Fifth Edition (WISC-V; Wechsler 61. The mean Intelligence Quotient for the 22q11.2DS group was 71.33 (SD = 13.41) and for the Down syndrome group was 65.84 (SD = 18.78), with no statistically significant difference between groups (t = 1.34, *p* > 0.05).

### Instruments

Language abilities were evaluated using a combination of standardized and widely used instruments to capture multiple domains of language. Receptive vocabulary was assessed with the Peabody Picture Vocabulary Test, Fourth Edition (PPVT-4; [23]), which measures the ability to understand spoken words through picture selection. Structural language skills, including morphology, syntax, and semantics, were evaluated using the Clinical Evaluation of Language Fundamentals – Fifth Edition (CELF-5; [63]), which provides standardized scores for core language, expressive and receptive language, and higher-order language skills. The BLOC-C (Bilingual Language Observation Checklist – Child version; adapted for English) was also administered to assess morphology, syntax, semantics, and pragmatics through clinician-administered observational tasks [52]. 

These assessment tools were chosen to provide complementary perspectives on language abilities. The PPVT-4 focuses on receptive vocabulary, providing a narrow but highly reliable measure of lexical knowledge. The CELF-5 offers a comprehensive evaluation of both receptive and expressive language across multiple structural and higher-order domains. The BLOC-C provides an observational and integrative measure across multiple linguistic domains, capturing both structural language and pragmatic abilities in a naturalistic context.

Although these batteries target overlapping aspects of language, they differ in methodology and scope. The PPVT-4 and CELF-5 rely on structured, standardized tasks with quantitative scoring, whereas the BLOC-C provides qualitative, observational data on multiple domains of language. This combination allows for a multifaceted analysis of language profiles, facilitating the identification of relative strengths and weaknesses within and across linguistic domains in 22q11.2DS deletion syndrome and Down syndrome. The inclusion of the BLOC-C also addresses the need for assessments that are interpretable by an international audience, as it captures both structural and functional aspects of language that may not be fully assessed by traditional standardized tests.

The following clinical indices were derived from the CELF-5 for statistical analysis: Receptive Language Index (RLI), Expressive Language Index (ELI), and Language Content Index (LCI). Additionally, the Peabody Picture Vocabulary Test (PPVT) was used as the primary measure for Receptive Vocabulary (RV). For the purpose of domain-specific comparisons, these indices are referred to collectively as the core linguistic domains.

### Procedure

Participants were recruited through national associations and clinics specializing in 22q11.2DS deletion syndrome and Down syndrome. Families were provided with detailed information about the study, and written informed consent was obtained prior to participation. All assessments were conducted in a quiet, child-friendly environment either at the research facility or at the participant’s school, depending on family preference and logistical considerations.

Upon arrival, participants first completed the Wechsler Intelligence Scale for Children – Fifth Edition (WISC-V; [61] to obtain a baseline measure of general cognitive ability. Following the IQ assessment, language evaluations were administered in a randomized order across participants to minimize order effects and fatigue. The PPVT-4 was used to assess receptive vocabulary, the CELF-5 measured structural and higher-order language abilities, and the BLOC-C was administered by a trained clinician to evaluate morphology, syntax, semantics, and pragmatic skills in structured but naturalistic tasks.

Each assessment session lasted approximately 90 to 120 min, with breaks provided as needed. All testing was conducted individually to ensure that performance reflected each participant’s abilities. Assessment scores were recorded immediately and later checked for accuracy by a second examiner. Families were debriefed at the end of the session, and participants received age-appropriate incentives for their participation.

This procedure ensured standardized administration across participants while maintaining flexibility to accommodate individual needs, thereby maximizing the reliability and ecological validity of the collected data.

### Data analysis

Data were analyzed using IBM SPSS Statistics (Version 28). Descriptive statistics were calculated for all measures. To test H1, independent-samples t-tests were used to compare the 22q11.2DS and Down syndrome groups on IQ and each language measure. Effect sizes (Cohen’s d) were computed to quantify the magnitude of group differences (d = 0.2 small, d = 0.5 medium, d = 0.8 large; Cohen 1988), with *p*-values reported to three decimal places and *p* < 0.001 applied when appropriate. To further examine differences in language profiles across domains, a mixed-design ANOVA was conducted with group (22q11.2DS vs. Down syndrome) as the between-subjects factor and language domain (morphology, syntax, semantics, pragmatics, and vocabulary) as the within-subjects factor. This analysis allowed us to assess both overall group differences and potential group × domain interactions.

To test H2, Pearson correlation coefficients were computed to examine the association between age and receptive vocabulary separately within each group. Differences between correlation coefficients were assessed using Fisher’s r—to—z transformation, allowing direct comparison of the strength of associations across groups.

To address H3, the pattern of performance across domains identified in the mixed ANOVA was examined to determine whether it reflected a dissociated profile in 22q11.2DS and a more homogeneous profile in Down syndrome.

All analyses were conducted using standardized scores provided by the assessment instruments. No additional z-standardization procedures were applied. Analyses were restricted to those directly relevant to the study hypotheses in order to ensure clarity and interpretability.

Data visualization techniques were used to support interpretation, including graphical representations of language performance across domains for each group and scatterplots illustrating the relationship between age and receptive vocabulary, with regression lines fitted separately for each group.

For the analyses of age–vocabulary associations, we used raw scores from the Peabody Picture Vocabulary Test (PPVT), as these are more sensitive to developmental variation across the studied age range.

## Results

### Group comparisons and normative benchmarks

Table 1 presents the comparison of means between Group 1 and Group 2. For the CELF indices, a normative benchmark of 100 (SD = 15) is included to provide context regarding the participants' performance relative to the general population.

**Table 1:** Language performance in 22q11.2DS and down syndrome groups

Measures	22q11.2DS	DS	t	p	Cohen’s d
Age	10.74 (3.41)	10.78 (2.82)	−0.04	.972	−0.01
Receptive Vocabulary	81.85 (21.37)	41.80 (21.07)	5.97	.001	1.89
Morphology	19.65 (8.68)	12.05 (8.06)	2.89	.006	0.91
Syntax	22.10 (11.92)	6.45 (5.15)	5.39	.001	1.70
Semantics	14.15 (4.79)	9.00 (4.72)	3.42	.002	1.08
Pragmatics	15.90 (7.92)	8.35 (5.15)	3.57	.001	1.13
Main Language Score (MLS)	79.45 (20.14)	44.70 (7.41)	7.24	.001	1.97
Receptive Language Index (RLI)	83.75 (22.29)	49.30 (6.45)	6.63	.001	1.83
Expressive Language Index (ELI)	80.30 (24.21)	48.90 (7.09)	5.56	.001	1.52
Linguistic Content Index (LCI)	87.25 (25.62)	49.80 (6.42)	6.33	.001	1.77
Linguistic Structure Index (LSI)	71.25 (15.48)	51.50 (10.11)	2.70	.019	0.73
Linguistic Memory Index (LMI)	85.58 (20.57)	44.21 (11.78)	6.40	<.001	1.82

*SD* Standard Deviation; Cohen’s d calculated to estimate effect size. All *p*-values are reported to three decimal places. Group 1 and Group 2 were matched for age. However, Group 1 significantly outperformed Group 2 across all language measures with very large effect sizes (d > 1.7). Both groups performed below the normative mean (100)

### Data visualization

**Fig. 1:**
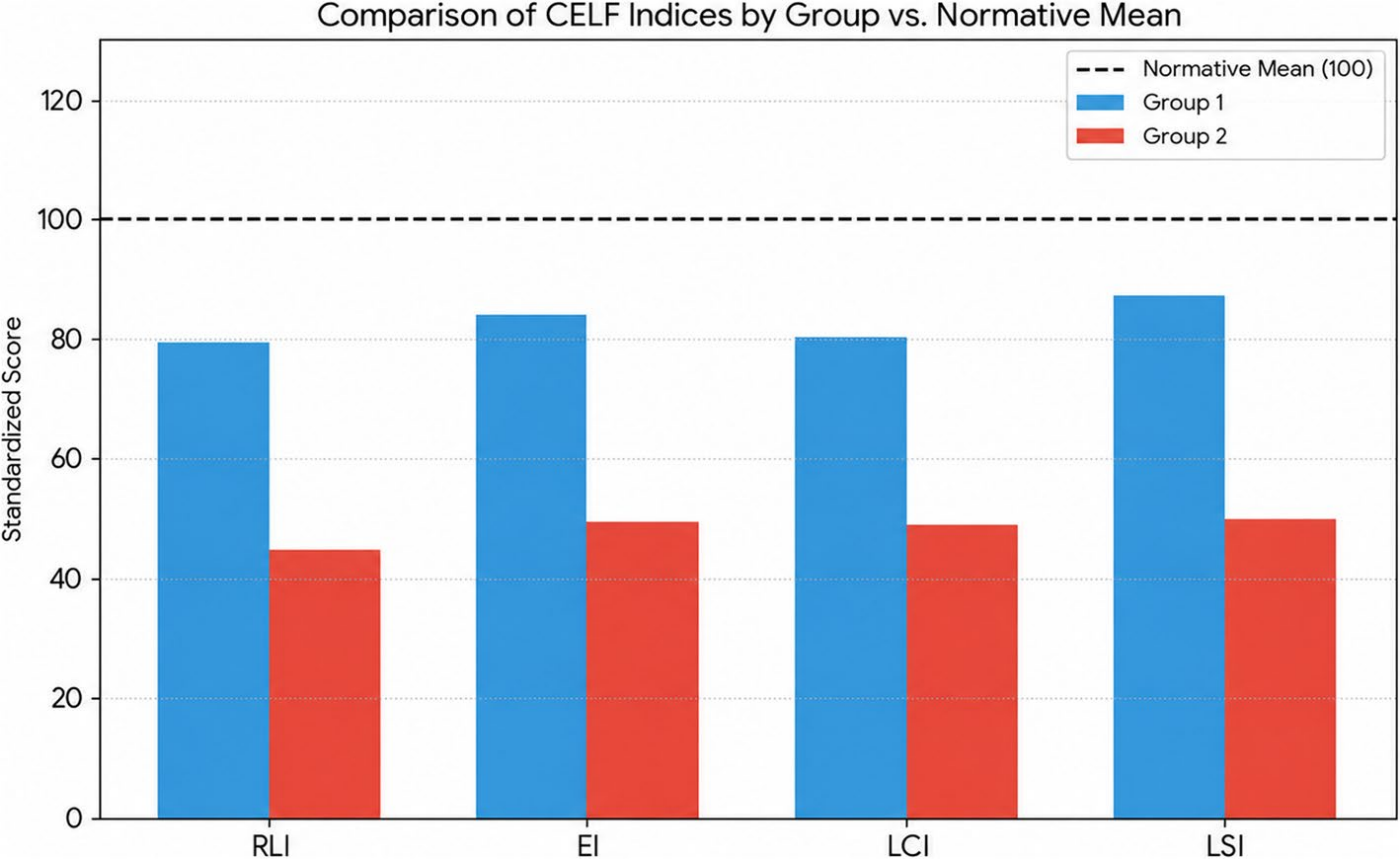
Language profiles for Group 1 and Group 2 across CELF-5 domains relative to normative benchmarks. Note: RLI (Receptive Language Index); ELI (Expressive Language Index); LCI (Language Content Index) and LSI (Linguistic Structure Index). All indices are based on standardized scores unless otherwise specified

To visualize the performance gap between groups and the normative population, Fig. 1 displays the standard scores for the CELF indices.

The visualization highlights that while Group 1 scores are closer to the normative range (though still below it), Group 2 scores are consistently and severely depressed across all linguistic domains, falling more than 3 standard deviations below the mean.

### Differences across language domains

To examine differences in language performance, a 2 × 4 mixed ANOVA was conducted with Group (22q11.2DS vs. Down syndrome) as the between-subjects factor and Domain (RV, RLI, ELI, LCI) as the within-subjects factor. A significant main effect of Group was observed, F(1, 78) = 163.29, *p* < 0.001, indicating that overall language performance differed substantially between groups. Across domains, the Down syndrome group performed at a markedly lower level than the 22q11.2DS group. The main effect of Domain was not significant, F(3, 78) = 1.04, *p* = 0.377, suggesting that, when averaging across groups, performance did not differ significantly between language domains.

Importantly, the Group × Domain interaction was not significant, F(3, 78) = 0.21, *p* = 0.889. This indicates that the pattern of performance across domains was broadly similar in both groups, with no evidence that one group showed a different configuration of relative strengths and weaknesses compared to the other. As illustrated in Fig. 1, both groups showed relatively parallel profiles, although at different levels of performance.

### Relationship between age and vocabulary

**Fig. 2:**
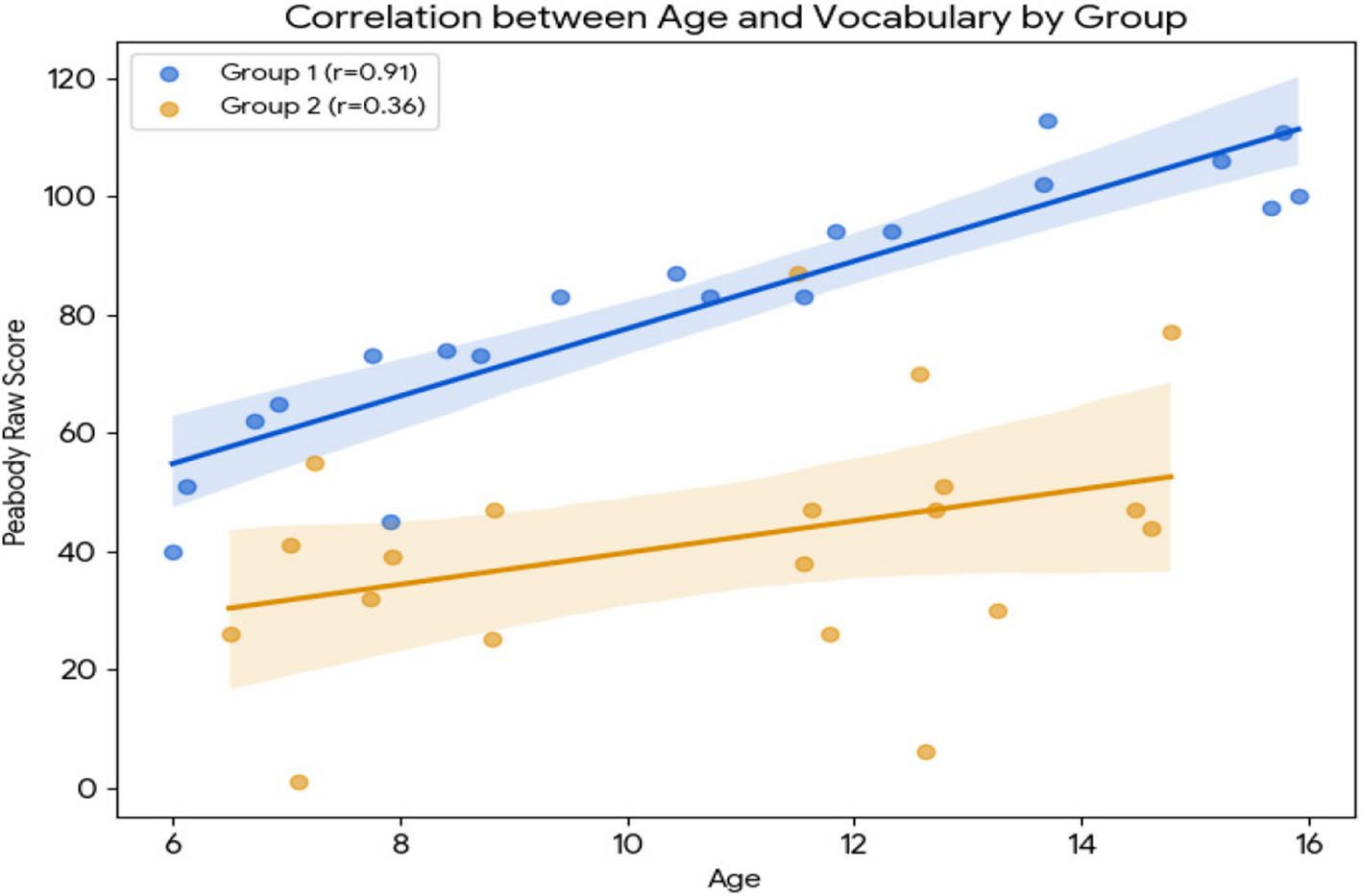
Scatter plot of the relationship between Chronological Age and Vocabulary (Peabody Raw Scores) by Group

To examine age-related differences in vocabulary performance, correlations between age and Peabody vocabulary scores were calculated. In the 22q11.2DS group, there was a strong positive correlation (r = 0.91, *p* < 0.001). In the Down syndrome group, the correlation was weaker and not statistically significant (r = 0.36, *p* = 0.120). A Fisher’s z-test confirmed that the difference between the two correlations was statistically significant (z = 3.37, *p* < 0.001), indicating that the strength of the age–vocabulary association differed reliably between groups (Fig. 2).

### General profile comparison

Hypothesis 3 largely restates the goals of Hypothesis 1. The results confirm that Group 2 exhibits a significantly lower and "flatter" linguistic profile than Group 1. The lack of a significant Interaction in the ANOVA and the significant difference in developmental correlations (H2) both support the conclusion that Group 2 is not just performing lower, but exhibits a lower level of performance across all domains.

## Discussion

The present study compared language performance in children and adolescents with 22q11.2 deletion syndrome (22q11.2DS) and Down syndrome (DS), with the aim of characterizing syndrome-related patterns of language functioning and their relation to age.

### Differences in overall language level

The primary finding is a marked difference in overall language performance between groups. Children with Down syndrome demonstrated substantially lower scores across all assessed domains compared to those with 22q11.2DS. This result is consistent with previous literature documenting more severe and pervasive language impairment in Down syndrome [2, 54, 55].

Crucially, however, the absence of a Group × Domain interaction indicates that this difference reflects a quantitative (severity-based) distinction, rather than qualitatively different language profiles. That is, both groups showed broadly similar patterns of performance across domains, differing primarily in overall level rather than configuration. This finding suggests that, within the domains assessed here, language abilities may be influenced by shared underlying constraints [34], with syndrome-related differences manifesting primarily as differences in degree rather than type.

Importantly, this does not preclude the existence of syndrome-related differences in language functioning, but suggests that, within the domains assessed here, such differences may operate primarily at the level of overall performance rather than in the structural organization of language. This interpretation is compatible with models proposing shared developmental constraints shaping language outcomes across neurodevelopmental conditions.

### Domain-level patterns and theoretical implications

Although the statistical analyses did not support the presence of distinct domain-specific profiles between groups (H3), descriptive patterns suggested certain relative trends across domains. Consistent with our analysis of the parallel profiles, both groups demonstrated a descriptive tendency toward higher scores in vocabulary relative to other domains. However, the lack of a significant Group × Domain interaction indicates that this pattern is shared across syndromes, rather than representing a syndrome-specific dissociation. This supports the notion of shared constraints on language organization despite the marked differences in overall severity (H1). For example, vocabulary tended to be relatively stronger compared to morphosyntactic performance, a pattern that has been reported in both populations [1, 10]. However, given the absence of statistically significant interaction effects, these patterns should be interpreted with caution and not as evidence of syndrome-specific configurations of language abilities. In 22q11.2DS, while vocabulary is often highlighted as a relative strength, it appears here to be aligned with the overall linguistic level when compared directly to the Down syndrome group.

In the case of 22q11.2DS, previous studies have consistently highlighted difficulties in pragmatic language use and contextualized communication (e.g., [32, 46]), even when structural aspects of language are relatively preserved. Similarly, although the Down syndrome group showed consistently low scores across domains, we do not interpret this as a statistically demonstrated "flat profile," but rather as a globally reduced level of performance across the assessed domains. We did not conduct specific within-group contrasts or variance-based profile analyses, which constrains further qualitative claims.

Rather than indicating clearly distinct language profiles, these findings may reflect relative variations within a broadly similar underlying structure of language abilities, consistent with the neuroconstructivist view of developmental disorders [19]. It is possible that more fine-grained measures—particularly in pragmatics, discourse-level processing, and functional communication—would be better suited to capturing subtle qualitative differences or dissociations between syndromes. Consequently, future research using ecologically valid assessments may be better positioned to detect syndrome-specific nuances that standardized batteries might overlook.

### Age-related differences in vocabulary

A key finding concerns the relationship between age and vocabulary performance. The strong positive association observed in the 22q11.2DS group suggests that vocabulary scores increase systematically with age in this group, a trend noted in studies highlighting their capacity for lexical accumulation [29]. In contrast, the weaker and non-significant association in the Down syndrome group indicates greater variability and a less consistent age-related pattern, which aligns with reports of early plateaus in language growth for this population [12, 15].

The significant difference between these correlations suggests that the age–language relationship differs between groups, with vocabulary development appearing more closely aligned with age in 22q11.2DS than in Down syndrome. However, these findings should be interpreted with caution. First, the data are cross-sectional and therefore do not permit direct inference about developmental trajectories. Second, the magnitude of the correlation in the 22q11.2DS group (r = 0.91) is unusually high, which may reflect methodological factors such as score scaling or restricted variability, as well as the use of raw scores which tend to inflate age associations in clinical samples [43].

### Implications for models of language development

The findings provide partial support for the use of genetically defined syndromes as models for examining constraints on language development [53]. The absence of profile differences suggests that domain-general constraints may play a substantial role in shaping language performance across syndromes. At the same time, differences in age-related patterns point to variation in developmental dynamics, which may reflect syndrome-specific influences on learning or cognitive processing. Thus, rather than indicating distinct language systems, the results are more consistent with shared organizational structures modulated by syndrome-related factors affecting overall level and developmental progression [59].

A primary limitation is the cross-sectional nature of the data, which prevents drawing conclusions about individual growth. As noted in the discussion, these findings represent age-related patterns rather than true developmental trajectories. Second, the absence of significant interaction effects constrains interpretation of domain-specific profile differences. Third, the use of standardized measures may not fully capture functional language use, particularly in pragmatics, which has been identified as a key area of difficulty in 22q11.2DS [25]. Finally, additional participant variables such as socioeconomic status, hearing status, and comorbidities were not extensively analyzed and may influence the generalizability of the findings.

In conclusion, this study demonstrates clear differences in overall language performance between children with 22q11.2DS and Down syndrome, alongside differences in the strength of age-related associations in vocabulary. However, there is no statistical evidence for distinct domain-specific language profiles between groups. These findings highlight the importance of distinguishing between differences in level and differences in structure when interpreting language performance in neurodevelopmental conditions.

## Data Availability

The datasets used and/or analysed during the current study are available from the corresponding author on reasonable request.
